# Factors associated with diagnostic delay in recurrent TB

**DOI:** 10.1186/s12889-020-09005-9

**Published:** 2020-08-08

**Authors:** Zhongyao Xie, Tingwei Wang, Hongguang Chen, Donglin Wang, Xiangqi Gao, Yi Hui

**Affiliations:** 1 Beijing Chest Hospital, Capital Medical University, Beijing, 101149 China; 2 Yulin Center for Disease Control and Prevention, Yulin, 719000 Shaanxi China; 3 Peking University Institute of Mental Health, National Clinical Research Center for Mental Disorders (Peking University Sixth Hospital), Key Laboratory of Mental Health, Ministry of Health (Peking University), No. 51 Hua Yuan Bei Road, Beijing, 100191 China

**Keywords:** Diagnostic delay, Recurrent tuberculosis, Survival analysis, Risk factor

## Abstract

**Background:**

Recurrent tuberculosis (TB) contributes to the burden of TB. The study was designed to explore the time of diagnostic delay and risk of delay in patients with recurrent TB in China.

**Methods:**

A total of 13,334 patients with new and recurrent TB registered in Yulin a city in China were included. The Kaplan-Meier survival curve was employed to estimate the median delay time. The mixed-effects survival model was used to identify the correlates associated with diagnostic delay. The outcome of interest in the model was"being diagnosed".

**Results:**

We found that 6.5% of cases with TB were attributed to recurrence. The median delay time of recurrent TB cases (73 days) was more than twice as long as that of new TB (35 days). Individuals with recurrent TB had a higher risk of diagnostic delay than new TB (HR, 0.5, 95%CI, 0.5–0.6). Factors associated with diagnostic delay differed between new TB and recurrent TB cases. Immigrants (HR, 0.5, 95%CI, 0.3–0.9), cases notified by way of recommendation (HR, 0.6, 95%CI, 0.4–0.9) and diagnosed at TB dispensary (HR, 0.4, 95%CI, 0.3–0.6) were associated with a higher risk of a longer delay for recurrent TB cases.

**Conclusions:**

The proportion of TB cases attributed to recurrence was high. Patients with recurrent TB had a longer delay time and a higher risk of diagnostic delay. Further interventions to improve diagnostic delay should focus on screening for TB in immigrants, improving public health services at the lowest healthcare level and update of TB diagnosis and management model.

## Background

Tuberculosis (TB) is still a threat to global public health, and a common cause of death. It was reported that 1.5 million people died of TB in 2018 [1]. Under the current global strategy for TB prevention and control, most first-episode TB patients can be treated effectively [2]. However, patients with TB who have received an adequate course of treatment might still develop a recurrent TB [3, 4]. Cases with recurrent TB may be infected with a new strain or be related to a relapse of an original infection [5–7]. The numbers and proportions of the patient with recurrent TB are important indicators to assess the effectiveness of TB control and proxies of TB drug-resistance [4]. A study performed in urban China reported that 5.3% of patients with TB had a recurrence after successful treatment, 18 times higher than the rate of TB in the general population [8]. According to previous studies, the 2-year incidence of recurrence after treatment of TB with rifampicin-containing regimens ranged from 0 to 27% [7]. Early detection, early diagnosis, and early treatment are the core strategies for TB control, which are also applicable to recurrent TB. Factors related to the delay in diagnosis and treatments of TB have been identified [2, 9], but the time of delay in diagnosis of recurrent TB cases and its correlates, to our knowledge, has not been well studied in China. The first aim of this study was, therefore, to compare the time of delay in patients with new and recurrent TB. The second was to estimate and compare the correlates associated with a longer delay in patients with new and recurrent TB. We hoped that the findings would illustrate the current situation of diagnostic delay for recurrent TB cases and provide information for early diagnosis and treatment.

## Methods

Yulin is located in the northernmost part of Shaanxi Province, China, consisting of 10 districts with over 3.1 million people. From Jan 1, 2008 to Dec 31, 2017, patients with new and recurrent TB registered in the city were enrolled in the study. The data included three types of information; individual (gender, age, household registration system, and occupation), the disease (onset time of TB symptoms, time of diagnosis and smear test results), and health care (ways of notification and type of diagnostic institution). Due to the time of the study and limitation of data, no information about HIV co-infection was obtained. The time delay in diagnosis was defined as the time from the onset of any TB symptom to the date of TB diagnosis. TB was diagnosed according to the standard definitions of the Guidelines for Implementing the National Tuberculosis Control Program in China. Medical Research Coordinating Committee of Yulin approved the study. Written informed consent forms were obtained from all subjects.

Recurrent TB disease occurred when patients who were previously treated for TB developed a new disease episode, due to either relapse (recurrence of the old infection) or reinfection (infection with a new strain).

According to the guidelines for TB control and prevention programs in China, TB sources were registered as notified by contact check, health examination, referral, active visit for symptoms, tracking and recommendation.

- “Referral” referred to when individuals were diagnosed as TB or presumptive TB in medical and health institutions (excluding TB dispensary) and then should be referred to local TB dispensary (Designated institute for local TB diagnosis and treatment management in China) for further diagnosis and treatment. Case notified by this route was registered as “Referral” in the TB registration system.
- “Active visit for symptoms”referred to when individuals themselves were aware that they might have suspicious symptoms of TB, and took the initiative to go to the local TB dispensary for diagnosis and treatment. Case notified by this route was registered as “Active visit for symptoms” in the TB registration system.
- “Tracking”referred to when individuals with TB and presumptive TB who were referred by medical and health institutions, however, did not go to the local dispensary on time, then local medical workers would track the cases.
- “Recommendation”referred to when individuals were identified as having suspicious symptoms of TB and recommended and urged to local TB dispensary for further diagnosis by medical workers or related personnel from the township, village or community health service center without diagnostic condition for TB.

### Data analysis and statistics

The median delay time was estimated by the Kaplan-Meier survival curve. A chi-square test and the log-rank test were used to compare the proportion and delay time separately. A two-level mixed-effects survival model was used to evaluate the hazard ratio (HR) and 95% confidence interval (95%CI) for predictors associated with the delay."being diagnosed" was regarded as the outcome of interest in the analysis. Therefore, predictors with HR less than 1 were regarded as risk factors. A two-sided P value < 0.05 was considered significant for all analyses. The database was constructed with EpiData v. 3.1 (EpiData Association, Denmark), and data was analyzed using SPSS v. (SPSS Inc., USA).

## Results

### Patient’s characteristics

A total of 13,334 TB cases were analyzed after excluding those under age of 15 years and with incomplete data records. Among them, 864 were identified was recurrent TB cases, accounting for 6.5% of the total. Significantly higher proportion of patients with recurrent TB were found in TB cases over 45 years, with local household registration, being farmers, notified by recommendation, tested smear positive, diagnosed in TB dispensary (*P*<0.05, see Table 1).

**Table 1:** Summary of demography and clinical characteristics of patients with recurrent TB (n = 864)

Variables	Recurrent TB (n)	Total (N)	Proportion (%)	*P*
Gender				
Male	517	8029	6.4	0.815
Female	347	5305	6.5	
Age				
≤ 45	304	7322	4.2	<0.001
> 45	560	6012	9.3	
Household				
Local	850	12,669	6.7	<0.001
Immigrant	14	665	2.1	
Occupation				
Others	207	4461	4.6	<0.001
Farmer	657	8873	7.4	
TB source				
Contact check	1	74	1.4	<0.001
Health examination	0	243	0.0	
Referral	166	3748	4.4	
Active visit for symptoms	495	6765	7.3	
Tracking	172	2182	7.9	
Recommendation	30	322	9.3	
Smear results				
Negative	462	9110	5.1	<0.001
Positive	402	4224	9.5	
Type of facilities for TB diagnosis				
Hospital	35	1510	2.3	<0.001
TB dispensary	829	11,824	7.0	

### Median delay time in the diagnosis of recurrent TB cases

**Fig. 1:**
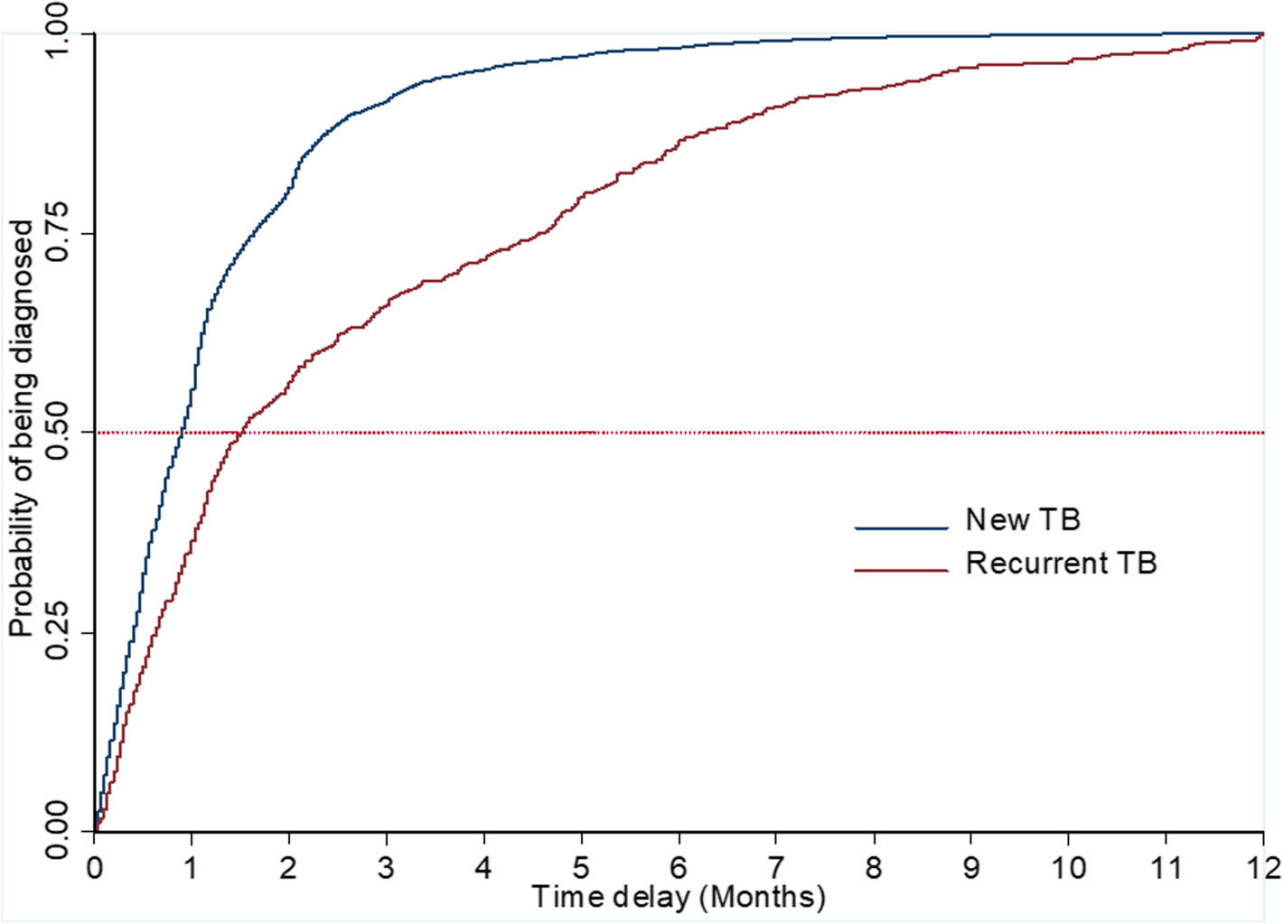
Estimation of diagnostic delay by Kaplan-Meier survival curve

The median delay time was 35 days (IQR,16–75) for all patients, 34 days (IQR,16–70) for cases with new TB, and 73 days (IQR, 25–243) for cases with recurrent TB. The median delay time of recurrent TB cases was more than twice as long as that of new TB, and the difference was statistically significant (log-rank test χ^2^ = 371.78, *P*<0.05, see Fig. 1). For new TB cases, significantly longer diagnostic delay was found among female gender, those ages above 45 years, immigrants, farmers, and cases notified by recommendation, smear positive, and those diagnosed in TB dispensary (*P*<0.05, see Table 2). The delay in diagnosis at the dispensary was longer for both new and recurrent cases, although the delay was only statistically significant for the recurrent cases (*P*<0.05).

**Table 2:** Median delay time stratified by new and recurrent TB

Variables	For new TB (IQR)	*P*	For recurrent TB (IQR)	*P*
Gender				
Male	33(16–68)	0.004	73(22–234)	0.680
Female	35(17–75)		72(27–250)	
Age				
≤ 45	32(15–64)	< 0.001	77(24–220)	0.511
>45	38(18–84)		65(25–249)	
Household				
Local	34(16–70)	< 0.001	72(24–243)	0.935
Immigrant	48(22–92)		119(48–182)	
Occupation				
Others	32(14–66)	< 0.001	118(30–266)	0.272
Farmer	35(17–73)		61(21–225)	
TB source				
Contact check	9(6–14)	< 0.001	–	0.079
Health examination	14(5–33)		–	
Referral	30(13–62)		35(12–168)	
Active visit	35(18–74)		74(25–300)	
Tracking	41(22–76)		128(36–201)	
Recommendation	67(26–140)		337(31–914)	
Smear results				
Negative	33(16–67)	< 0.001	92(26–220)	0.115
Positive	38(18–86)		59(21–272)	
Type of facilities for TB diagnosis				
Hospital	31(14–69)	0.011	25(16–57)	< 0.001
TB dispensary	34(17–71)		75(26–254)	

### Correlates associated with longer diagnostic delay for recurrent TB cases

Three mixed-effects survival models were used in this study. In the first model, all the TB cases were considered as a whole, and the classification of TB (new and recurrent TB) was included as the independent variable and other variables as covariant. Then we performed two separate mixed-effects survival models stratified by new and recurrent TB to explore correlates associated with diagnostic delay within each group. In model 1, recurrent TB was found to be associated with increasing the risk of diagnostic delay (HR, 0.5, 95%CI, 0.5–0.6, see Table 3). In model 2, correlates associated with diagnostic delay in new TB cases were consistent with that in model 1. In model 3, only immigrants (HR, 0.5, 95%CI, 0.3–0.9), cases notified by recommendation (HR, 0.6, 95%CI, 0.4–0.9) and diagnosed at TB dispensary (HR, 0.4, 95%CI, 0.3–0.6) were found to be associated with a higher risk of a longer delay.

**Table 3:** Factors associated with diagnostic delay of recurrent TB^†^

Variables	Model 1 (With all subjects)	Model 2 (New TB only)	Model 3 (Recurrent TB only)
	HR(95%CI)	HR(95%CI)	HR(95%CI)
Gender, Female	0.9(0.9–0.9)**	1(0.9–1)*	1(0.9–1.1)
Age, >45	0.8(0.8–0.9)***	0.8(0.7–0.8)***	0.9(0.8–1.1)
Household, Immigrant	0.8(0.7–0.9)***	0.8(0.7–0.9)***	0.5(0.3–0.9)*
Occupation, Famer	1.0(0.9–1.0)	1(0.9–1)	1(0.9–1.2)
TB source, Health examination	0.6(0.5–0.8)***	0.7(0.5–0.9)**	–
TB source, Referral	0.4(0.3–0.5)***	0.4(0.3–0.5)***	–
TB source, Active visit	0.3(0.2–0.4)***	0.3(0.3–0.4)***	0.9(0.8–1.1)
TB source, Tracking	0.3(0.2–0.4)***	0.4(0.3–0.5)***	1.1(0.9–1.4)
TB source, Recommendation	0.2(0.2–0.3)***	0.2(0.2–0.3)***	0.6(0.4–0.9)*
Smear results, Positive	0.9(0.8–0.9)***	0.8(0.8–0.8)***	0.9(0.8–1.1)
Type of facilities for diagnosis, TB dispensary	0.9(0.8–0.9)***	0.9(0.8–0.9)***	0.4(0.3–0.6)***
TB classification, Recurrent TB	0.5(0.5–0.6)***	–	–

Note: “†” In the two-level ((level 1- individuals and level 2-dispensaries) mixed-effects survival model analysis among all cases and new cases, both the estimate of variance and the standard error was less than 0.01. An LR test comparing the model with the one-level survival model did not favour the random-intercept model with *P* > 0.05. However, among the recurrent TB cases, the estimate of variance in level 2 was 0.14 with the standard error of 0.07 and the LR test comparing the model with the one-level survival model favoured the random-intercept model with *P*<0.05

“*” *P*<0.05, “**” *P*<0.01, “***” *P*<0.001

## Discussion

This study explored the delay time and factors associated with diagnostic delay among recurrent TB cases in China. We found that 6.5% of TB cases were attributed to relapse or exogenous reinfection after treatment completion or cured. It was reported that at least 5.3% of the notified TB patients experienced one or more recurrences of TB in Shanghai of China [8]. Recurrent TB accounted for 1.3% of all cases in Barcelona [10] and 4.1% in England and Wales [11]. The proportion of TB cases attributable to relapse or exogenous reinfection in different settings should be carefully compared because there were differences in enrollment criteria, patient follow-up periods, and study design. These findings intensified the evidence that TB patients with completed treatment or cured were still at high risk for another episode. We found that the time of diagnostic delay for recurrent TB cases was more than twice that of new cases. Meanwhile, recurrent TB doubled the risk of delay when compared with new TB cases. Recurrent TB contributes to ongoing transmission of infection to contacts of cases in the home, community, and health facilities. In theory, people who have had TB before should be familiar with the symptoms of the disease. When they are re-infected and show symptoms, they should have a shorter diagnostic delay than cases with new onset of TB. However, the results of this study found that this was not the case. Therefore, further study on exploring the underlying reasons for the longer diagnostic delay of recurrent TB cases is of great significance for TB control. Consistent with the results of a study from Nepal [12], smear positive cases had a longer diagnostic delay than smear negative cases. This might be related to the unbalanced medical resources available to smear negative and smear positive cases, or to the lead time bias caused by that smear negative cases might be found to have TB because of other diseases. Further research is needed to address this issue. Population-based reports on risk factors associated with diagnostic delay of recurrent TB cases are lacking. In this study, the association between immigrants and diagnostic delay among patients with recurrent TB differed in univariate and multivariate analysis. This might be related to the confounding factors in univariate analysis. It can be seen from HRs that immigration had a greater impact on the diagnostic delay of recurrent tuberculosis compared to that of a new case. Previous studies had identified immigrant as an independent risk factor for recurrence [13]. According to China’s Migrant Population Development Report in 2018, there were about 244 million immigrants in China by 2017. There were many barriers to prevent the immigrant population from seeking help in a timely fashion. A study from eastern China reported that interventions to improve health-seeking behaviors among the immigrant population must focus on strengthening their labor, medical security, and health education [14]. “Referral”,“Active visit for symptoms”,“Tracking”, and “Recommendation”were common passive case finding (PCF) pathways which remained the main way to detect TB cases in countries with high TB burden [15]. As indicated in this study, more than 90% of TB cases were notified by PCF. This was particularly true for people with recurrent TB, with almost 100% notified by PCF. It is well known that cases notified by PCF will suffer a longer diagnostic delay than those notified by active case finding (ACF) [16]. This could partly explain why people with recurrent TB have longer delays. Unlike the results analyzed in model 1 and model 2, the only recommendation was associated with a longer diagnostic delay in model 3. This phenomenon also reflected that the identification and recommendation of TB patients or presumptive cases still need to be improved at the community level [17]. Shortage of trained health providers and TB knowledge at primary health care facilities were important causes of diagnostic delay, as well as factors related to the health facility staff’s inability to refer TB suspects to county TB dispensaries or designated hospitals for TB care and misdiagnosis [9, 18]. Similar to findings from previous studies [19, 20], a longer diagnostic delay was reported among cases diagnosed with TB in TB dispensary than in designed hospitals. Cases with presumptive TB symptoms commonly visited hospitals first, therefore they could be diagnosed in the same place if they had TB, which could shorten patient care pathways. At present, China is also promoting the integration model with TB ‘designated’ hospital responsible for diagnosis and treatment and TB dispensary for the public health service aspects of TB control [21].

The current study has two potential limitations. On one hand, information on the onset date of TB symptoms was recalled by the patient, and recall bias could not be avoided, which might lead to overestimation among recurrent TB cases and underestimation among new TB cases. On the other hand, TB prevention and control strategies and measures varied in different settings, and extrapolation of local findings needed to be cautious.

## Conclusions

In summary, our findings showed that a high proportion of TB cases attributed to relapse or exogenous reinfection after treatment completion or cured was found in northwest China. The diagnostic delay time and risk of delay were significantly longer and higher in cases with recurrent TB than those with new TB. Immigrants, cases notified by way of recommendation, and those diagnosed at TB dispensary were factors associated with increasing the risk of diagnostic delay of cases with recurrent TB. Interventions to improve the diagnostic delay among recurrent TB may focus on strengthening the health education after cured or treatment completion, management of TB cases among the immigrant population and improving the ability of TB notification and referral at the primary health care facilities as well as promoting the integration model highlighting the role of the designated hospital in TB diagnosis.

## Data Availability

The data that support the findings of this study are available from Yulin Center for Disease Control and Prevention. Still, restrictions apply to the availability of these data, which were used under license for the current study, and so are not publicly available. Data are however available from the authors upon reasonable request and with permission of Yulin Center for Disease Control and Prevention.

## Abbreviations

Tuberculosis: TB
Hazard Ratio: HR
95% Confidence Interval: 95%CI
Interquartile Range: IQR
Passive Case Finding: PCF
Active Case Finding: ACF
